# Increased IL-22- and IL-17A-Producing Mucosal-Associated Invariant T Cells in the Peripheral Blood of Patients With Ankylosing Spondylitis

**DOI:** 10.3389/fimmu.2018.01610

**Published:** 2018-07-13

**Authors:** Éric Toussirot, Caroline Laheurte, Béatrice Gaugler, Damien Gabriel, Philippe Saas

**Affiliations:** ^1^INSERM CIC-1431, University Hospital of Besançon, Clinical Investigation Center in Biotherapy, Besançon, France; ^2^Fédération Hospitalo-Universitaire INCREASE, University Hospital of Besançon, Besançon, France; ^3^University Hospital of Besançon, Department of Rheumatology, Besançon, France; ^4^Université Bourgogne Franche-Comté, Department of Therapeutics and EPILAB EA4266: “Epigenetique des infections virales et des maladies inflammatoires”, Besançon, France; ^5^EFS Bourgogne Franche-Comté, INSERM CIC-1431, Plateforme de BioMonitoring, Clinical Investigation Center in Biotherapy, Besançon, France; ^6^Sorbonne Universités, UPMC Univ Paris 06, INSERM UMR938, Centre de Recherche Saint-Antoine (CRSA), Paris, France; ^7^AP-HP, Hôpital Saint-Antoine, Service d’Hématologie Clinique et Thérapie Cellulaire, Université Paris 06, Paris, France; ^8^Neurosciences intégratives et cliniques EA 481, Univ. Franche-Comté, Univ. Bourgogne Franche-Comté, Besançon, France; ^9^INSERM U1098, Etablissement Français du Sang Bourgogne Franche Comté, Université Bourgogne Franche-Comté, Interactions Hôte-Greffon-Tumeurs, LabEx LipSTIC, Besançon, France

**Keywords:** ankylosing spondylitis, IL-17A, IL-22, mucosal-associated invariant T cells, mucosal immunity

## Abstract

The IL-23/T helper 17 (Th17) axis plays an important role in joint inflammation in ankylosing spondylitis (AS). Conventional CD4^+^ Th17 cells are a major source of IL-17A. IL-22 is another cytokine implicated in AS pathophysiology and is produced by Th17 and Th22 cells. In this study, we aimed to analyze conventional and non-conventional T cell subsets producing IL-17A and IL-22 in patients with AS. We thus evaluated the intracellular staining for IL-17A, IL-22, and IFN-γ in peripheral blood mononuclear cells of 36 patients with AS and 55 age- and sex-matched healthy controls (HC). Conventional CD4^+^ and CD8^+^ T cells, γδ T cells, and mucosal-associated invariant T (MAIT) cells were evaluated. In patients with AS, we found a decreased frequency and number of γδ T cells, of MAIT cells and of IFN-γ^+^ CD4^+^ and CD8^+^ T cells. Th17-related IL-17A^+^/IFN-γ^−^ CD4^+^ T cells were decreased in AS. The number of IL-22^+^ MAIT cells was higher in AS compared with HC, as well as the number of IFN-γ^+^/IL-17A^+^ MAIT cells. The number of IFN-γ^−^/IL-17A^+^ MAIT cells was higher only in female patients with AS compared with female HC. The cellular source of IL-17A was thus not restricted to conventional Th17 CD4^+^ T cells and might involve innate-like T cells, such as MAIT cells. Circulating MAIT cells producing IL-22 were increased in AS. These results strengthen the importance of innate and innate-like sources of IL-17A and/or IL-22.

## Introduction

Ankylosing spondylitis (AS) is the prototypical and most common form of spondyloarthritis (SpA). The IL-23/T helper 17 (Th17) axis has been suggested to be a key player in joint inflammation in AS (1, 2). Indeed, AS has been found to be associated with a genetic polymorphism of the IL-23 receptor (*IL-23R*) gene (3), and IL-23 is a maturation and growth factor for IL-17A-secreting cells. HLA-B27, a major genetic predisposing factor for AS, has the property to be misfolded during the assembly of its heavy chain in the endoplasmic reticulum, and this phenomenon generates IL-23 production (4). Circulating IL-17A^+^ CD4^+^ T cells and IL-17A-producing γδ T cells are expanded in AS, together with increased serum concentrations of IL-23 and IL-17A (5). Moreover, IL-23 overexpression in a mouse model of arthritis induces a SpA-like disease characterized by enthesitis with an inflammatory infiltrate containing IL-23R^+^ resident T cells expressing retinoid acid receptor-related orphan receptor γt, and these cells produce IL-17A and IL-22 (6). Finally, therapeutic trials targeting IL-17A have been proven to be effective in patients with AS (7).

It is estimated that around 50% of patients with AS exhibit microscopic signs of ileum or colon inflammation and an average of 6.5% of SpA patients develop clinically established inflammatory bowel disease, highlighting the link between gastrointestinal tract inflammation and SpA (8). New cellular players, as a source of IL-23 and/or IL-17A, have recently been identified in AS, and some of these cells are present in mucosal tissues, such as the gut (9). They include innate-like T cells including mucosal-associated invariant T (MAIT) cells and innate lymphoid cells, most of which have the capacity to produce IL-17A and related effector cytokines, such as IL-22. Recently, it has been shown that MAIT cells may be implicated in AS by contributing to the production of IL-17A (10, 11). IL-22 is a member of the IL-10 cytokine family that is produced by different types of lymphocytes including activated Th17 cells and Th22 cells (12). It is considered that IL-22 has dual functions, protective or pathogenic, in inflammatory conditions depending on the stage of the disease (13). Thus, identifying cells that produce the relevant cytokines in AS (i.e., IL-17A and/or IL-22) is a key challenge. In this study, we aim to analyze conventional and non-conventional T cell subsets producing IL-17A and IL-22.

## Patients and Methods

### Patients and Healthy Controls (HC)

Thirty-six patients with AS and 55 sex-matched and age-matched HC were included in the study. All the AS patients met the modified New York criteria and were all under nonsteroidal anti-inflammatory drugs and/or conventional synthetic disease-modifying anti-rheumatic drugs (sulfasalazine or methotrexate). None of the patients had corticosteroids or had previously received a TNF inhibitor (most of them were enrolled before initiating such agent). The study was approved by the local Ethics Committee (*Comité de Protection des Personnes CPP-EST-II*, Besançon, France), and the sample collection was approved by the French Ministry of Higher Education and Research (agreement number #DC-2008-713).

### Methods

Absolute numbers of blood T cells, CD4^+^ and CD8^+^ T cells were on fresh samples determined by single platform flow cytometry using the TetraCXP^®^ method, Flow-Count fluorospheres, and FC500^®^ cytometer (Beckman Coulter, Villepinte, France) according to the manufacturer’s recommendations, as previously described (14). Peripheral blood mononuclear cells (PBMCs) were isolated from peripheral blood by centrifugation using a Ficoll-Hypaque gradient and were then slowly frozen and stored in liquid nitrogen. Sample freezing allows us to perform staining and flow cytometry analysis in batches. This minimizes variation between samples. Surface phenotype and intracellular staining was thus performed after slowly defrosted. Stained cells were analyzed by multi-color flow cytometry using a FACS Canto II cytometer (BD Biosciences, Le Pont de Claix, France) and Kaluza1.3 software (Beckman Coulter, Villepinte, France). Antibodies used were FITC-conjugated CD8, PE-TCRγδ (Beckman Coulter), PECy7-CD161, APCCy7-TCRVα7.2 (BioLegend, Ozyme, Saint-Quentin-en-Yvelines, France), V450-CD4, and V500-CD3 (BD Biosciences). For intracellular cytokine detection, PBMCs were stimulated with 50 ng/mL phorbol myristate acetate (Sigma-Aldrich, Saint Quentin Fallavier, France) and 1 µg/mL ionomycin (Sigma-Aldrich) for 6 h or left unstimulated. GolgiPlug (1/1,000, BD Biosciences) was added 5 h before the end of stimulation. Then, cells were stained with Fixable viability Dye (FVD506, eBioscience, Paris, France) to exclude dead cells and surface stained with PE-CD3, APC-CD4 (Beckman Coulter), PercpCy5.5-CD8 (eBioscience), and APCCy7-TCRVα7.2 (BioLegend). Then, cells were fixed and permeabilized with Cytofix/Cytoperm (BD Biosciences) and stained with FITC-IL-17A (R&D Systems), PE-Cy7-IFN-γ (BD Biosciences), and efluor 450-IL-22 (eBioscience). Data were collected on a FACS Canto II and analyzed with Kaluza software.

Serum IL-17A, IL-22, and TNF-α levels were determined by ELISA according to the manufacturer’s instructions (Kit BioLegend, Ozyme).

Erythrocyte sedimentation rate (ESR) test and C-reactive protein (CRP) levels were used as laboratory parameters to assess inflammation.

### Statistical Analysis

Results are expressed as mean ± SEM. Absolute number of the different T cell subsets, cytokine-secreting T cell subsets, and MAIT cells was obtained by multiplying the specific cell subset percentages by the total CD3^+^ lymphocyte number. These values were obtained by extrapolation of CD4^+^, CD8^+^, and MAIT T cells by using the absolute numbers of T cells which were obtained on fresh blood samples and the percentages of the specific subpopulations among CD3^+^ T cells obtained in thawed samples. The differences in the number of the evaluated cellular subsets, cytokine-secreting T cell subsets, and MAIT cells between patients with AS and HC were analyzed using the non-parametric Mann–Whitney test. Spearman’s correlation coefficient was used to test the correlation between disease activity and the percentage of cytokine-secreting MAIT cells and between IL-22-positive cells and circulating IL-22.

## Results

**Figure 1:**
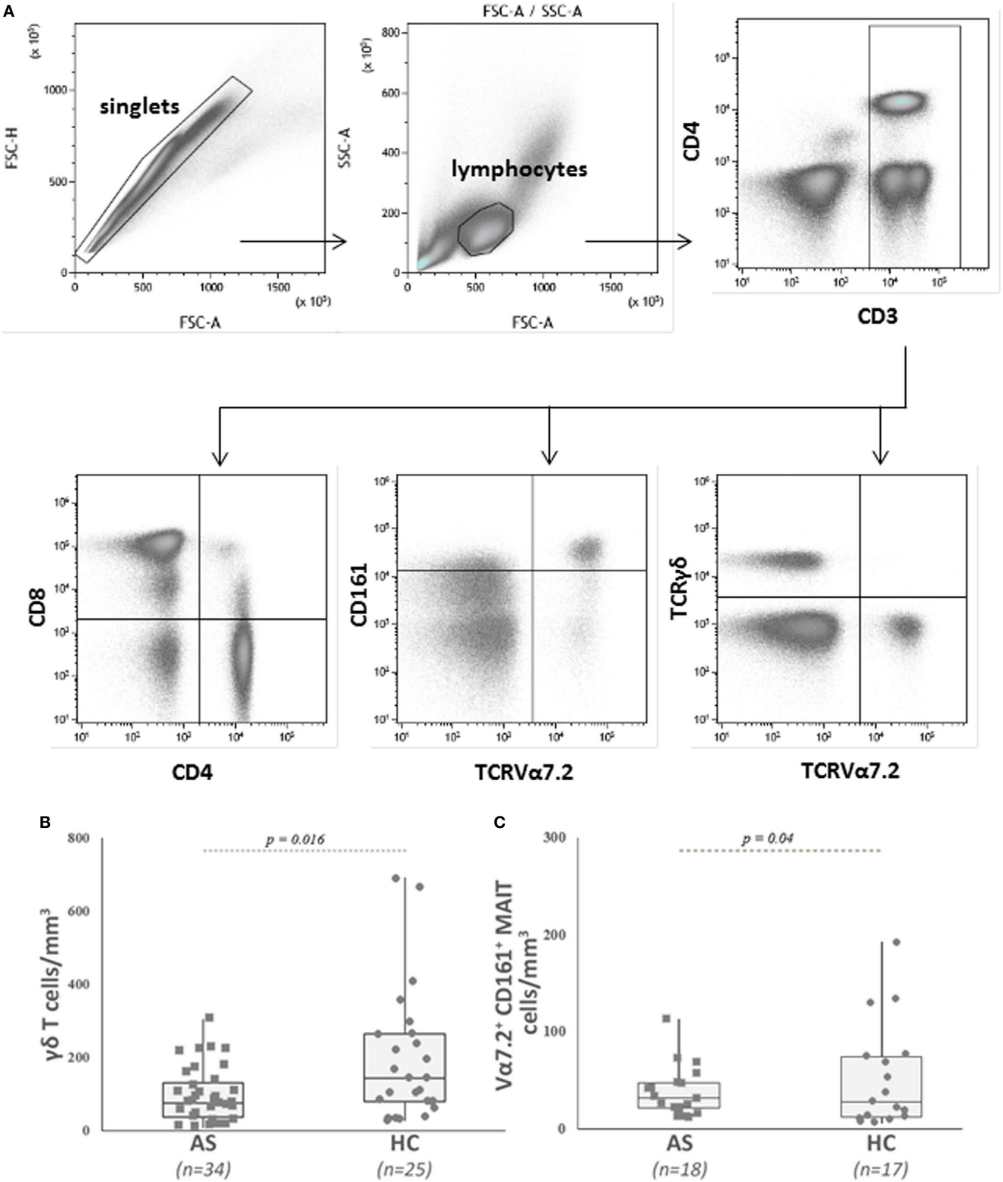
Analysis of blood γδ T cells and mucosal-associated invariant T (MAIT) cells in patients with ankylosing spondylitis (AS) and in healthy controls (HC). **(A)** Representative dot plots showing the gating strategy used to identify conventional CD4^+^ and CD8^+^ T cells, MAIT, and γδ T cells in peripheral blood mononuclear cell by flow cytometry. CD3^+^ T cells were gated on total lymphocytes (FSC/SSC plot after doublet exclusion), then MAIT cells were identified as TCRVα7.2 CD161^high^ among CD3^+^ T cells. TCRγδ^+^ T cells were identified among CD3^+^ T cells. Of note, MAIT cells are included in the CD8^+^ T cell population (see Table 2 for explanations). **(B)** AS patients showed a significant lower number of circulating γδ T cells than HC. Circulating γδ T cells were analyzed as described in Section “Patients and Methods” and gated as described in **(A)**. Absolute numbers (cells/mm^3^) were obtained by multiplying the percentages of γδ T cells by the total T lymphocyte number. Each symbol represents an AS patient (squares, *n* = 34) or HC (circles, *n* = 25). **(C)** AS patients showed a significant lower number of circulating MAIT cells than HC. Circulating MAIT cells were analyzed as described in Section “Patients and Methods” and gated as described in **(A)**. Absolute numbers (cells/mm^3^) were obtained by multiplying the percentages of MAIT cells by the total T lymphocyte number. Data are depicted as box plots (the band inside the box corresponds to the median). Each symbol represents an AS patient (squares, *n* = 18) or HC (circles, *n* = 17).

The demographics and clinical characteristics of the patients and HC are described in Table 1. Patients had active disease according to Bath ankylosing spondylitis disease activity index (BASDAI) and ankylosing spondylitis disease activity score (ASDAS) and had higher CRP levels compared with HC (*p* = 0.008). The absolute number and percentage of CD4^+^ T cells, CD8^+^ T cells, and CD3^+^ T cells did not differ between patients and HC (Table 2). On the contrary, we observed a lower number and proportion of γδ T cells and Vα7.2^+^ CD161^+^ MAIT cells in patients with AS compared with HC (*p* = 0.016 and *p* = 0.04, respectively) (Table 2; Figures 1A–C).

**Table 1:** Demographic, clinical, and biological characteristics as well as circulating IL-22, IL-17A, and TNF-α levels of patients with AS (*n* = 36) and HC (*n* = 55).

	AS (*n* = 36)	HC (*n* = 55)	*P*
Age (years)	44.7 ± 2.6	47.7 ± 1.8	0.3
Sex (male/female)	27/9	32/23	0.1
Disease duration (years)	12.7 ± 1.6		
Extra articular manifestations	*n* = 5		
	*n* = 9		
Psoriasis	*n* = 2		
Uveitis			
Inflammatory bowel disease			
Conventional synthetic DMARDs	*n* = 1		
	*n* = 1		
Sulfasalazine			
Methotrexate			
BASDAI	5.4 ± 0.3		
ASDAS	3.3 ± 0.2		
BASFI	4.6 ± 0.4		
HLA B27	31		
ESR (mm/h)	24.1 ± 4.5	11.4 ± 2.6	0.1
CRP (mg/L)	11.9 ± 3.4	6.5 ± 2.7	0.008
IL-22 (pg/mL)	22.6 ± 8.4	15.1 ± 4.4	0.7
IL-17A (pg/mL)	0.23 ± 0.1	0.007 ± 0.007	0.0009
TNF-α (pg/mL)	3.67 ± 1.7	0.9 ± 0.05	0.002

*BASDAI, Bath ankylosing spondylitis disease activity index; BASFI, Bath ankylosing spondylitis functional index; ASDAS, ankylosing spondylitis disease activity score; ESR, erythrocyte sedimentation rate; CRP, C-reactive protein; AS, ankylosing spondylitis; HC, healthy controls; DMARDs, disease-modifying anti-rheumatic drugs*.

**Table 2:** T cell subsets, cytokine-secreting helper T cells, and mucosal-associated invariant T (MAIT) cells in patients with ankylosing spondylitis (AS) (*n* = 36) and healthy controls (HC) (*n* = 55).

	AS	HC	p*
CD3^+^ T cells/mm^3^ (%)	1,479.7 ± 91.7 (54.1 ± 3.3) *n* = 36	1,669.8 ± 103.5 (53.8 ± 2.3) *n* = 28	0.25
CD4^+^ T cells/mm^3^ (%)	781.05 ± 68.5 (50.2 ± 2.2) *n* = 34	683.6 ± 86.1 (47.1 ± 2.5) *n* = 25	0.35
CD8^+^ T cells/mm^3a^ (%)^a^	625.9 ± 41.7 (42.2 ± 2.2) *n* = 34	708.1 ± 59.8 (38.9 ± 1.9) *n* = 25	0.3
γδ T cells/mm^3^ (%)	95.9 ± 13 (6.5 ± 0.8) *n* = 34	194 ± 36.1 (10.4 ± 1.2) *n* = 25	**0.016**
Vα7.2^+^ CD161^+^ MAIT cells/mm^3^ (%)	38.7 ± 6.2 (2.6 ± 0.4) *n* = 18	52.4 ± 13.2 (4.5 ± 0.8) *n* = 17	**0.04**

**Cytokine-secreting conventional T cell subsets**			
IFN-γ^+^ CD4^+^ T cells/mm^3^ (%)	33.05 ± 4.9 (4.6 ± 0.7) *n* = 34	47.9 ± 10.3 (13.1 ± 1.6) *n* = 25	**<0.0001**
IFN-γ^+^ CD8^+^ T cells/mm^3a^ (%)^a^	155.8 ± 18.1 (23.3 ± 1.9) *n* = 34	261.3 ± 31.1 (38.3 ± 2.2) *n* = 25	**<0.0001**
IL-17A^+^/IFN-γ^+^ CD4^+^ T cells/mm^3^ (%)	0.028 ± 0.01 (0.005 ± 0.002) *n* = 36	0.016 ± 0.006 (0.004 ± 0.006) *n* = 27	0.68
IL-17A^+^/IFN-γ^+^ CD8^+^ T cells/mm^3a^ (%)^a^	0.07 ± 0.03 (0.01 ± 0.04) *n* = 36	0.26 ± 0.14 (0.022 ± 0.008) *n* = 28	0.37
IL-17A^+^/IFN-γ^−^ CD4^+^ T cells/mm^3^ (%)	0.29 ± 0.08 (0.04 ± 0.008) *n* = 36	0.38 ± 0.2 (0.16 ± 0.03) *n* = 28	**0.0005**
IL-17A^+^/IFN-γ^−^ CD8^+^ T cells/mm^3a^ (%)^a^	0.11 ± 0.03 (0.017 ± 0.003) *n* = 36	0.4 ± 0.2 (0.04 ± 0.01) *n* = 28	0.11
IL-22^+^/IFN-γ^+^ CD4^+^ T cells/mm^3^ (%)	0.67 ± 0.25 (0.11 ± 0.04) *n* = 36	0.6 ± 0.3 (0.10 ± 0.04) *n* = 28	0.7
IL-22^+^/IFN-γ^+^ CD8^+^ T cells/mm^3a^ (%)^a^	4.7 ± 3 (0.52 ± 0.3) *n* = 36	2 ± 0.9 (0.19 ± 0.06) *n* = 28	0.4
IL-22^+^/IFN-γ^−^ CD4^+^ T cells/mm^3^ (%)	0.9 ± 0.16 (0.14 ± 0.02) *n* = 36	0.9 ± 0.2 (0.13 ± 0.07) *n* = 28	0.32
IL-22^+^/IFN-γ^−^ CD8^+^ T cells/mm^3a^ (%)^a^	0.38 ± 0.08 (0.07 ± 0.02) *n* = 36	0.5 ± 0.13 (0.07 ± 0.01) *n* = 28	0.5

**MAIT cells according to intracellular cytokine staining**			
IFN-γ^+^ MAIT cells/mm^3^ (%)	9.29 ± 1.8 (22.2 ± 2.1) *n* = 18	13 ± 3.5 (25.2 ± 1.9) *n* = 17	0.8
IFN-γ^+/^IL-22^+^ MAIT cells/mm^3^ (%)	0.37 ± 0.09 (1.1 ± 0.3) *n* = 18	0.11 ± 0.03 (0.32 ± 0.09) *n* = 17	**0.02**
IFN-γ^−^/IL-22^+^ MAIT cells/mm^3^ (%)	0.36 ± 0.09 (0.83 ± 0.1) *n* = 18	0.07 ± 0.06 (0.29 ± 0.06) *n* = 17	**0.002**
IFN-γ^−^/IL-17A^+^ MAIT cells/mm^3^ (all) (%)	0.14 ± 0.05 (0.43 ± 0.1) *n* = 18	0.037 ± 0.02 (0.24 ± 0.05) *n* = 17	**0.01**
IFN-γ^−^/IL-17A^+^ MAIT/mm^3^ (males) (%)	0.78 ± 0.09 (0.34 ± 0.5) *n* = 11	0.013 ± 0.01 (0.28 ± 0.05) *n* = 4	0.29
IFN-γ^−^/IL-17A^+^ MAIT/mm^3^ (females) (%)	0.23 ± 0.1 (0.57 ± 0.2) *n* = 7	0.04 ± 0.03 (0.15 ± 0.1) *n* = 13	**0.037**
IFN-γ^+^/IL-17A^+^ MAIT/mm^3^ (all) (%)	0.08 ± 0.05 (0.17 ± 0.07) *n* = 18	0.04 ± 0.03 (0.14 ± 0.05) *n* = 17	**0.06**
IFN-γ^+^/IL-17A^+^ MAIT/mm^3^ (males) (%)	0.02 ± 0.03 (0.05 ± 0.02) *n* = 11	0.013 ± 0.01 (0.01 ± 0.02) *n* = 4	**0.027**
IFN-γ^+^/IL-17A^+^ MAIT/mm^3^ (females) (%)	0.18 ± 0.1 (0.35 ± 0.17) *n* = 7	0.045 ± 0.04 (0.15 ± 0.14) *n* = 13	**0.01**

*Results [mean ± SEM] are given as absolute number of cells per mm^3^ (cells/mm^3^) and percentage (%). The number (*n*) of subjects who were evaluated for each cell subset is also given. Absolute number of the different T cell subsets, cytokine-secreting T cell subsets, and MAIT cells was obtained by multiplying the specific cell subset percentages by the total lymphocyte number*.

*p Values are given for comparison of absolute number of T cells subsets between AS and HC. Statistically significant data are written in bold font.

*^a^Of note, MAIT cells which express the CD8 marker are included in the analyzed CD8^+^ T cell population. MAIT cells are not excluded, since they represent a low percentage of circulating CD8^+^ T cells compared with conventional CD8^+^ T cells (2.6 ± 0.4 or 4.5 ± 0.8% MAIT cells vs. 42.2 ± 2.2 or 38.9 ± 1.9% total CD8^+^ T cells) in AS patients or HC, respectively*.

**Figure 2:**
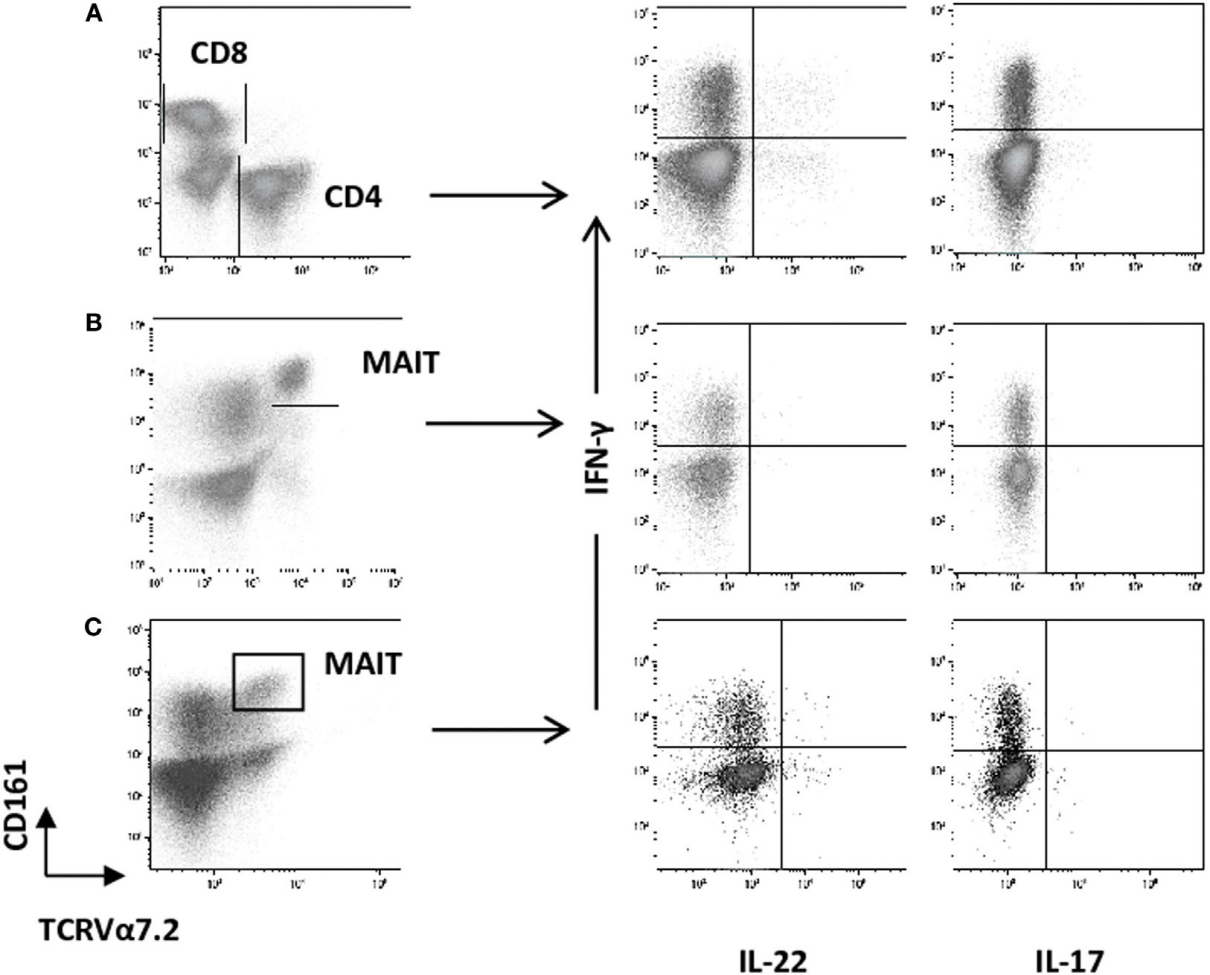
Representative dot plots showing the gating strategy used to measure intracellular cytokines among CD4^+^ and CD8^+^ T cells and mucosal-associated invariant T (MAIT) cells from healthy controls (HC) and ankylosing spondylitis (AS) patients by flow cytometry. Cells were activated *ex vivo* as described in Section “Patients and Methods” and stained for the detection of intracellular cytokines. CD3^+^ T cells were gated on total lymphocyte (FSC/SSC plot after doublet exclusion), then MAIT cells were identified as TCRVα7.2 CD161^high^ among CD3^+^ T cells. Intracellular IL-22, IL-17A, and IFN-γ are quantified among CD4^+^ T cells **(A)** and MAIT cells **(B,C)**, respectively [**(A)** vs. **(B,C)**]. IL-22, IL-17A, and IFN-γ are depicted among MAIT cells for a HC **(B)**, or an AS patient **(C)**.

**Figure 3:**
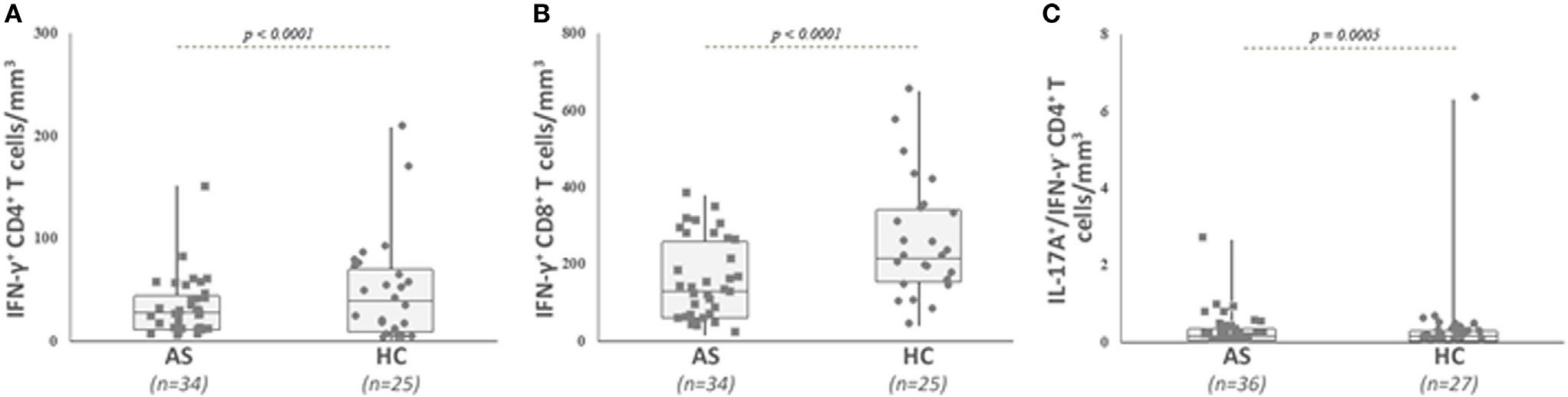
Analysis of IFN-γ secreting conventional CD4^+^ and CD8^+^ T cells, as well as IL-17A- and IFN-γ-producing CD4^+^ T cells in patients with ankylosing spondylitis (AS) and healthy controls (HC). Circulating cytokine-secreting conventional T cells were analyzed as described in Section “Patients and Methods” and gated as described in Figure 2. Absolute numbers (cells/mm^3^) were obtained by multiplying the percentages of these T cells by the total T lymphocyte number. AS patients exhibited a significant lower number of IFN-γ secreting conventional CD4^+^ T cells **(A)**, IFN-γ secreting conventional CD8^+^ T cells **(B)**, and IL-17A positive IFN-γ negative CD4^+^ T cells **(C)** than HC. Data are depicted as box plots (the band inside the box corresponds to the median). Each symbol represents an AS patient (squares, *n* = 34 or 36) or HC (circles, *n* = 25 or 27).

We then analyzed the intracellular expression of IL-17A, IFN-γ, and IL-22 in different T cell subpopulations (Figures 2A–C). First, we observed that Th1-related IFN-γ^+^ CD4^+^ T cell and IFN-γ^+^ CD8^+^ T cell counts were significantly decreased in the AS group (Table 2; Figures 3A,B) (both *p* value <0.0001). Then, we found that the following CD8^+^ T cell subsets: IL-17A^+^/IFN-γ^+^, IL-17A^+^/IFN-γ^−^, IL-22^+^/IFN-γ^+^, and IL-22^+^/IFN-γ^−^ were in equal proportions in both groups. Thus, no difference exists for CD8^+^ T cells, except a decrease in IFN-γ^+^ Tc1 cells in AS patients.

Concerning CD4^+^ T cell subsets, only the number of IL-17A^+^/IFN-γ^−^ cells was decreased in AS compared with HC (Table 2; Figure 3C) (*p* = 0.0005), while the other cytokine-secreting CD4^+^ T cell subsets did not differ between patients and HC (Table 2).

**Figure 4:**
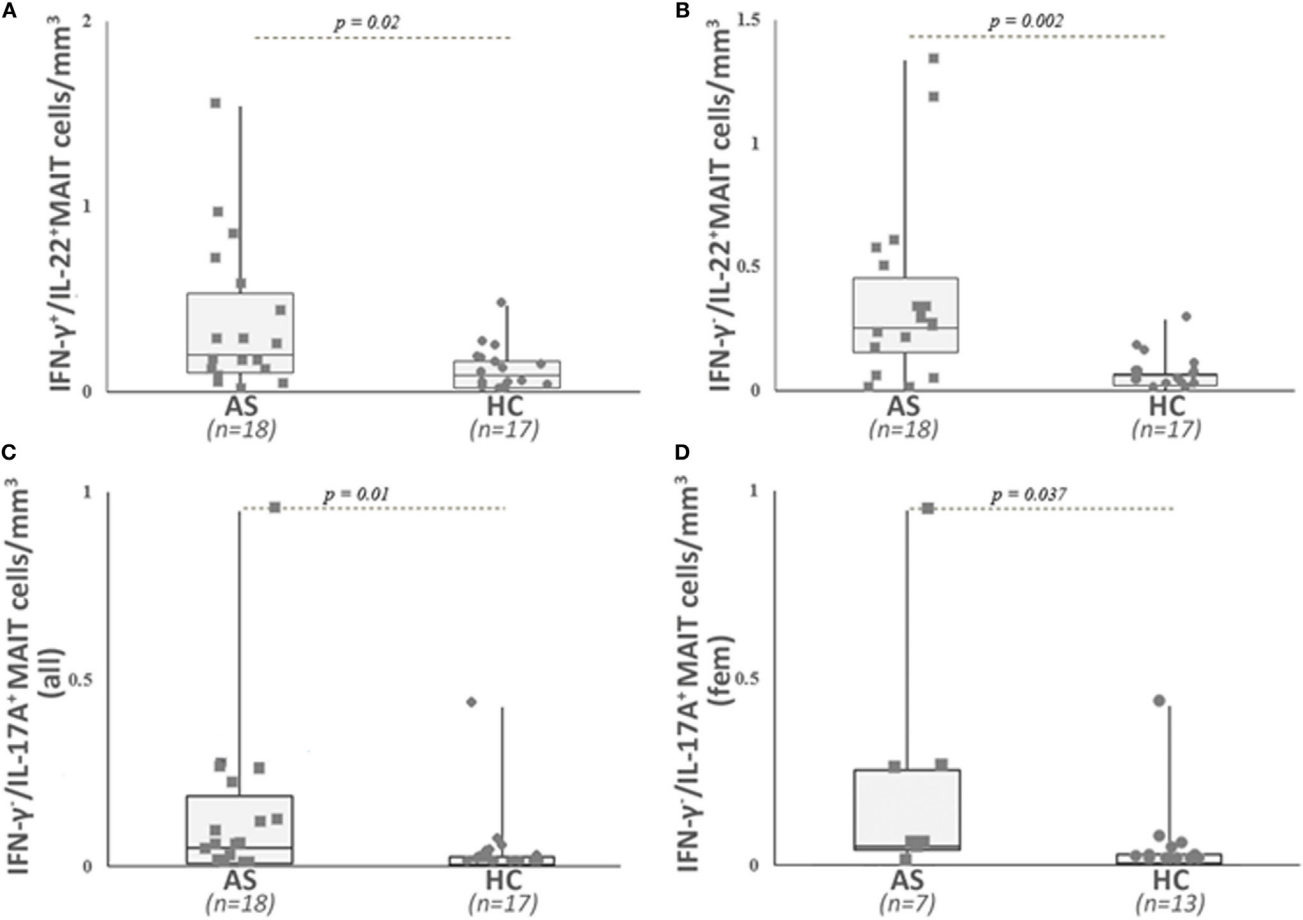
Analysis of different cytokine-secreting mucosal-associated invariant T (MAIT) cells in patients with ankylosing spondylitis (AS) and in healthy controls (HC). Circulating cytokine-secreting mucosal-associated invariant T (MAIT) cells were analyzed as described in Section “Patients and Methods” and gated as described in Figure 2. Absolute numbers (cells/mm^3^) were obtained by multiplying the percentages of these cytokine-secreting MAIT cells by the total T lymphocyte number. AS patients exhibited a significant higher number of IL-22- and IFN-γ-secreting MAIT cells **(A)**, IFN-γ-negative IL-22-positive MAIT cells **(B)**, and IFN-γ negative IL-17A-positive MAIT cells **(C)** than HC. IFN-γ-negative IL-17A-positive MAIT cell counts are found increased only in female AS patients when compared with healthy women **(D)**. Data are depicted as box plots (the band inside the box corresponds to the median). Each symbol represents an AS patient (squares, *n* = 18 or 7) or HC (circles, *n* = 17 or 13).

When examining the MAIT population, we observed a significant higher number of IL-22-producing cells in the patient group (IFN-γ^+^/IL-22^+^ and IFN-γ^−^/IL-22^+^ MAIT cells: *p* = 0.02 and *p* = 0.002, respectively) (Table 2; Figures 4A,B), as well as a higher number of IL-17A producing MAIT cells (IFN-γ^−^/IL17A^+^ and IFN-γ^+^/IL-17A^+^ MAIT cells: *p* = 0.01 and 0.06, respectively) compared with HC (Figure 4C; Table 2). Since a sexual dimorphism was described for IL-17A-producing cells (15), we also examined the results for IL-17A^+^ MAIT cells according to the gender. IFN-γ^+^/IL-17A^+^ MAIT cell counts were increased in both male and female AS compared with male and female HC (*p* = 0.027 and *p* = 0.01). For the MAIT cells that were only positive for IL-17A (i.e., IFN-γ^−^/IL-17A^+^ MAIT), a significant higher frequency was observed only in female AS (*p* = 0.037) (Figure 4D; Table 2).

Finally, serum levels of IL-22 were found marginally elevated in patients with AS, albeit without reaching a statistical significance (22.6 ± 8.4 vs. 15.1 ± 4.4 pg/mL in AS vs. HC, *p* = 0.7). Other circulating inflammatory cytokines (i.e., IL-17A and TNF-α) were significantly higher in the AS group (*p* = 0.0009 and *p* = 0.002, respectively), but their levels were very low (Table 1). No correlation was found between disease activity (BASDAI, ASDAS, and CRP) and percentage of IL-17A^+^ or IL-22^+^ MAIT cells. We observed also no correlation between circulating IL-22 and IL-22^+^ MAIT cells (all *p* > 0.05).

## Discussion

In addition to the prototypical inflammatory cytokine TNF-α, the IL-23/Th17 axis is currently considered to play a central role in driving inflammation in AS (1, 2). However, only few studies have characterized the cells that are responsive to IL-23 and/or produce IL-17A and IL-22 in the peripheral blood of patients with AS, and at sites of disease inflammation. We found that AS patients are characterized by normal absolute numbers or frequencies of conventional CD4^+^ T cells and CD8^+^ T cells, but that they had a decreased frequency of circulating γδ T, MAIT, and IFN-γ^+^ Th1 cells. We did not observe a higher frequency of IL-17A^+^ Th17 or IFN-γ^+^/IL-17A^+^ Th1/Th17 cells in our AS patients. Indeed, in our study, AS patients were characterized by decreased frequencies of IL-17A^+^/IFN-γ^−^ CD4^+^ (Th17) cells. In a previous study, the proportion of IL-23R-expressing T cells (i.e., cells potentially producing IL-17A) in the periphery was twice as high in AS patients than in HC, but this increased frequency was only observed in the γδ T cell subset that represented the main source of IL-17A secretion (16). The proportion of “conventional” CD4^+^ T cells that were positive for IL-17A were found in a lower proportion in AS patients compared with the frequency detected in the control population (16). These data fit well with our findings. By contrast, another study assessing the phenotype of Th17-secreting cells in AS found that conventional CD4^+^ T cells were the primary producers of IL-17A (17). These cells were increased in the peripheral blood of patients with AS (17). Similar findings were observed in another study with an increased proportion and absolute number of circulating IL-17A^+^/IFN-γ^−^ Th17 cells in patients with AS (18). All these previous works restricted the assessment of intracellular IL-17A expression in the CD4^+^ T cell lineage and γδ T cells (16–18). The circulating CD8^+^ T cell subset was not systematically examined; only unpublished data were reported by Kenna et al. (16) with unchanged proportions of IL-17A^+^ CD8^+^ T cells. We studied this subset and found also no difference in AS patients when compared with HC. The discrepancies observed between these studies (16–18) and our results may be related to the patient characteristics, the disease status and/or its duration, and the treatments received by the AS patients. Indeed, corticosteroids and TNF-α inhibitors may potentially modulate the number of Th17 cells and the levels of circulating IL-17A (19). On the contrary, a previous work has shown that the IL-23/Th17 axis is not influenced by TNF inhibitors, since after 24-month administration of these agents in patients with AS, IL-23 remained unchanged and stayed elevated while in parallel a clinical improvement was observed (20). In addition, in the study by Hayashi et al. (11), 15 of 30 patients with AS received a TNF inhibitor, but MAIT cell frequency or IL-17A production by MAIT cells did not differ between patients who received the biological agent and those who did not. In our study, no patient had corticosteroids and/or TNF-α inhibitors, and thus, concomitant treatments could not have biased our results.

We observed a lower proportion of γδ T cells and Th1 cells in our patients with AS. Limited data are available on this cellular subset in AS. Decreased proportions of CD4^+^ and CD8^+^ T cells secreting IFN-γ and TNF-α corresponding to Th1 and Tc1 cells, respectively, have been previously described in AS, and also in healthy HLA-B27-positive control subjects (21), suggesting that AS can be considered as a low IFN-γ disease. As stated above, an enrichment of circulating IL-17A-secreting γδ T cells was reported in AS with, in parallel, a normal proportion of IL-17A^+^ CD4^+^ and CD8^+^ T cells (16). This supports the idea that IL-17A may come from another source than conventional Th17 cells. Presumably, a migration of γδ T cells to inflammatory sites may also account for a reduced number of this cell subset in the blood of patients. In support of this hypothesis, γδ T cells were found to accumulate in the enthesitis in a mouse model of SpA-like disease that overexpressed IL-23 (22).

Th22 is a CD4^+^ T cell subset characterized by the production of a distinct profile of effector cytokines, namely, IL-22, TNF-α, and IL-13, but not IL-17A (12, 23). Since it has been shown that IL-22 is produced by Th22 and also Th17, this cytokine and related cellular sources may be relevant to the pathogenesis of AS. We found that the numbers of IL22^+^ CD4^+^ and IL22^+^ CD8^+^ T cells did not differ between AS and HC. Previous works have demonstrated that Th22 or IL-22 is deregulated in AS. Indeed, in the study by Zhang et al., the percentage and number of Th22 cells were increased in patients with AS and correlated with plasma IL-22 levels (18). Another study reported similar findings, with an increased frequency of IL-22^+^ CD4^+^ T cells in patients with AS (17). Again, the inconsistencies between these results and ours may be explained by the patient characteristics and/or potentially, the received treatments.

Mucosal-associated invariant T cells are innate-like lymphocytes preferentially present but not exclusively in the gut lamina propria (24). They are implicated in different autoimmune diseases, such as rheumatoid arthritis (RA) and multiple sclerosis (24). In a mouse model of arthritis, it was demonstrated that MAIT cells may exacerbate arthritis, suggesting an arthritogenic potency (25). While we observed a decreased proportion of MAIT cells in AS patients, MAIT cells positive for IL-22 were found to be elevated in our patients. Moreover, IFN-γ^+^/IL-17A^+^ MAIT cells were upregulated in patients with AS, while IFN-γ^−^/IL-17A^+^ were also found increased but only in female patients. Few data are available concerning the frequency and function of MAIT cells in AS. One recent study found a reduced frequency of MAIT cells in the peripheral blood of patients with AS, while the synovial fluid was enriched with this cell population (10). In addition, this work found a higher frequency of IL-17A^+^ MAIT cells, especially in male AS patients. Finally, priming MAIT cells by IL-7 was associated with an upregulation of intracellular IL-17A, a result that was not observed with IL-23 (10). In another study, MAIT cells in the peripheral blood were found at a lower frequency compared with HC, but these cells had the capacity to produce high levels of IL-17A (11). In another study, the frequency of MAIT cells did not differ between patients with SpA, RA, fibromyalgia, and HC, albeit a tendency to a decreased frequency was observed in the SpA group. However, CD8^+^ MAIT cells were increased in patients with SpA compared with HC (26). Finally, another analysis did not find any difference in the number and frequency of circulating of MAIT cells between patients with AS and HC (27). Collectively, the results by Gracey et al. (10) and Hayashi et al. (11) are in line with ours, highlighting the potential role of MAIT cells in AS, and their capacity to produce IL-17A and IL-22. They also support the hypothesis that the source of IL-17A is not restricted to canonical CD4^+^ Th17 cells. We also evaluated more precisely the MAIT cytokine producing population by the assessment of double cytokine staining, i.e., IFN-γ and IL-17A. We observed in our study the potency of MAIT cells to produce IL-22. The pathogenic role of IL-22 has previously been reported in certain autoimmune diseases, such as psoriasis (23). The role of IL-22 in arthritis has been suggested in a collagen-induced arthritis model, in which IL-22-deficient mice exhibited decreased incidence of arthritis and less pannus formation (28). In addition, it has been suggested that IL-22 may promote osteoclastogenesis in RA (12). In SKG mice that developed a SpA disease after microbial antigen injection, IL-22 together with IL-17A have been shown to promote peripheral enthesitis (29). In parallel, in a mouse model of SpA, overexpression of IL-23 induces the production of IL-22 by entheseal IL-23R^+^ CD4^−^/CD8^−^ CD3^+^ T cells, and IL-22 was associated with the induction of genes (e.g., *Col2a* or *Acan*) coding for factors that regulate bone formation and promote ligamentous ossification, a hallmark of AS disease (6). IL-22, together with IFN-γ and TNF-α, has also the capacity to influence the proliferation and differentiation of mesenchymal stroma cell (MSC) toward an osteogenic phenotype. IL-22 alone can promote the expression of osteogenic transcription factors (e.g., *RUNX2, ALPL*, and *COL1A1*), indicating that these effects on MSC may represent a pathway for new bone formation in AS (30). Type 3 innate lymphoid cells (ILC3) are another source of IL-22 that have been found to be expanded in the gut, peripheral blood and bone marrow of patients with AS, and these cells also produced IL-17A (31). Finally, copy number variations of the *IL-22* gene have been associated with AS in the Chinese population with a low copy number as a being protective factor for AS (32).

Our study has some limitations. We did not evaluate all the circulating cells that produce IL-17A, such as ILC3 or invariant natural killer T cells, neither IL-22-secreting NK22 cells. Analysis is only performed in the blood compartment, because our patients had exclusive axial disease, without peripheral arthritis and joint effusion. Our patient group included a limited number of women (especially for the IFN-γ- and IL-17A-secreting MAIT cell analysis), but this was also found in previous studies analyzing IL-17A-secreting cells (16–18). Analysis of cytokine-secreting cells was performed on frozen cells that may have impaired our results. However, sample freezing allows us to perform staining and cytometry analysis in batches: minimizing variation between samples. The HC group was adequately age- and sex matched. On the contrary of previous works evaluating MAIT cells (10, 11) or IL-17A-secreting cells in AS (16–18), our patients did not receive a biological agent that could influence the results.

In conclusion, our results indicate that the cellular source of IL-17A is not restricted to conventional CD4^+^ Th17 cells, suggesting that the type-17 axis may be broader in AS than initially thought. Besides the IL-23 and IL-17A cytokines, IL-22 also seems to be implicated in the pathogenesis of AS. MAIT cells are deregulated in AS, strengthening the importance of innate and innate-like sources of IL-17A and IL-22 and emphasizing the link between the gut and the joint in AS. Future studies are needed to better understand the importance of this cellular population and IL-22-producing cells in AS.

## Ethics Statement

Ethics committee: Comité de Protection des Personnes CPP-EST-II, Besançon, France. Sample collection was approved by the French Ministry of Higher Education and Research (agreement number #DC-2008-713).

## Author Contributions

ET, BG, and PS conceived and designed the study. ET recruited the patients and healthy controls. BG and CL designed the laboratory assessments and performed all the analysis. ET and BG analyzed the results. ET wrote the manuscript together with BG and PS. DG designed the figures. All the authors contributed substantially to editing the manuscript and approved the final version.

## Conflict of Interest Statement

The authors declare that the research was conducted in the absence of any commercial or financial relationships that could be construed as a potential conflict of interest.
